# Self-adhesive hydrogel meshes reduce tissue incorporation and mechanical behavior versus microgrips self-fixation: a preclinical study

**DOI:** 10.1007/s10029-021-02552-z

**Published:** 2022-01-07

**Authors:** Selma Benito-Martínez, Marta Rodríguez, Francisca García-Moreno, Bárbara Pérez-Köhler, Estefanía Peña, Begoña Calvo, Gemma Pascual, Juan Manuel Bellón

**Affiliations:** 1 Departamento de Medicina y Especialidades Médicas, Facultad de Medicina y Ciencias de la Salud, Universidad de Alcalá, 28805 Alcalá de Henares, Madrid Spain; 2 Biomedical Networking Research Centre On Bioengineering, Biomaterials and Nanomedicine (CIBER-BBN), 28029 Madrid, Spain; 3 Ramón y Cajal Health Research Institute (IRYCIS), 28034 Madrid, Spain; 4 Departamento de Cirugía, Facultad de Medicina y Ciencias de la Salud, Ciencias Médicas y Sociales, Universidad de Alcalá, 28805 Alcalá de Henares, Madrid Spain; 5 Aragón Institute of Engineering Research (I3A), Universidad de Zaragoza, Zaragoza, Spain

**Keywords:** Hernia repair, Self-adhesive meshes, Self-gripping meshes, Tissue integration, Biomechanical test

## Abstract

**Purpose:**

Atraumatic mesh fixation for abdominal hernia repair has been developed to avoid the disadvantages of classical fixation with sutures, which is considered a cause of chronic pain and discomfort. This study was designed to analyze, in the short and medium term, the biological and mechanical behavior of two self-fixing meshes compared to that of a polypropylene (PP) mesh fixed with a cyanoacrylate (CA) tissue adhesive.

**Methods:**

Partial abdominal wall defects (6 × 4 cm) were created in New Zealand rabbits (*n* = 36) and repaired using a self-adhesive hydrogel mesh (Adhesix™), a self-gripping mesh (ProGrip™) or a PP mesh fixed with CA (Surgipro™ CA). After 14 and 90 days, the host tissue incorporation, macrophage response and biomechanical strength were examined.

**Results:**

At 14 and 90 days, the ProGrip and Surgipro CA meshes showed good host tissue incorporation; however, the Adhesix implants presented poor integration, seroma formation and a higher degree of shrinkage. The Adhesix hydrogel was completely reabsorbed at 14 days, whereas ProGrip microhooks were observed at all study times. The macrophage response was higher in the ProGrip and Surgipro CA groups at 14 and 90 days, respectively, and decreased over time. At 90 days, the ProGrip implants showed the highest tensile strength values and the Adhesix implants showed the highest failure stretch.

**Conclusion:**

Meshes with mechanical microgrip self-fixation (ProGrip) show better biological and mechanical behavior than those with adhesive hydrogel (Adhesix) in a preclinical model of abdominal hernia repair in rabbits.

## Introduction

The implantation of prosthetic materials for the repair of hernial defects in the abdominal wall is one of the most frequent general surgical procedures [1]. Different meshes for hernia repair have evolved in recent years in terms of the structure and chemical composition [2–6]. However, polypropylene (PP) meshes continue to be the most often used material for abdominal hernia repair.

These PP reticular prostheses, in the surgical implant procedure, when necessary, are usually fixed to the surrounding tissues using penetrating devices, to prevent migration, which can lead to hernia recurrence.

The short-term strength of mesh fixation is an undescribed factor in hernia repair but could have significant implications for early recurrence and mesh contraction [7]. Numerous techniques are available for fixation, and the most frequently used classical fixation devices range from simple sutures to different stapling systems as well as tacks at the laparoscopic level [8–11]. These routinely used fixation devices have significantly reduced the recurrence rates for hernia repair; however, they are time-consuming and led to postoperative discomfort and chronic pain, which have a significant impact on health-related quality of life [12].

In this respect, many researchers recommend reducing or avoiding the use of these classical devices to reduce the traumatic effects at the tissue level; thus, tissue adhesives have become popular and prostheses with self-adhering properties have been developed [13–16].

Nonpenetrating mesh fixation technologies have many advantages, such as easy handling and speed of use, reducing operative time [17]. Specifically, any method of fixation that does not use sutures or tacks to penetrate the underlying tissue where it is implanted has continued to gain popularity because such methods minimize the tissue trauma caused by sutures when the needle or tack is inserted, which represents an important consideration, especially in anticoagulated patients [18]. Finally, these methods can prevent certain adverse effects, including irritation, entrapment or injury to nerve endings [13], which in some cases would explain the postoperative pain of patients undergoing hernioplasty [19–21].

Mesh–tissue interfacial strength can also be used as a marker of mesh–tissue integration in the long term or mesh–tissue adhesion in the short term. Either marker indicates the capacity of the mesh–tissue complex to brace or carry mechanical loading, which is a critical mechanism for absorbing the stresses that develop in the abdominal wall under physiological loading conditions in both the short and long term [7]. Therefore, these novel adhesive strategies must also be strong enough to provide adequate clamping force to prevent the mesh from becoming detached, mainly in the short term before host tissue ingrowth.

The most widely used tissue adhesives specifically for hernia repair are fibrins of biological origin [22, 23]; however, long-chain cyanoacrylate-type tissue adhesives, for which toxicity problems have been minimized, are increasingly being used and show excellent biocompatibility and mechanical behavior and are currently authorized for use in clinical practice [24, 25].

In the group of atraumatic fixation devices, two relatively novel interesting prostheses, which have not been very widely tested, incorporate absorbable microhooks (ProGrip) or adhesive hydrogel (Adhesix) into the traditional PP prosthesis, thus allowing for self-fixing without any other additional element; thus, their advantages may lead to promising outcomes [26, 28].

Taking into account all these aspects, the aim of the present preclinical study was to analyze the biological and mechanical behavior of these two self-fixing meshes relative to that of a PP mesh fixed using a synthetic adhesive (n-hexyl) cyanoacrylate (CA).

## Materials and methods

### Experimental animals and meshes

**Fig. 1:**
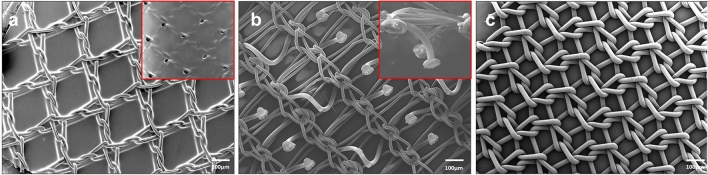
Characterization of the prosthetic materials. Scanning electron microscopy (SEM) images (15 × magnification) of the Adhesix (**a**), Progrip (**b**) and Surgipro (**c**) meshes. Further magnified views of the reabsorbable layer of Adhesix (20×) and reabsorbable microhooks of Progrip (40×) are shown in the box

The maintenance of the animals used in this study and the experimental procedures were carried out in accordance with the current protocols on the use of animals in experimentation (European Directive 2010/63/EU, European Convention of the Council of Europe ETS123 and Spanish Royal Decree 53/2013), and the study was approved by the Animal Experimentation Ethics Committee of Universidad de Alcalá (Spain). In this study, partial defects were created in the abdominal wall in a total of 36 New Zealand white rabbits weighing 3 kg, and the animals were divided into three groups according to the prosthesis used in the repair. Two study times at 14 and 90 days were established. Each of the groups had n = 6 animals. The materials used to repair the defects were two self-fixing meshes or a Surgipro mesh fixed with a CA adhesive for medical use (Fig. 1):

*Adhesix™* (Bard DAVOL Inc., Warwick, RI, USA) is a self-adhesive mesh composed of a PP mesh covered by a layer of polyethylene glycol and polyvinylpyrrolidone, and in the presence of moisture, this layer forms a hydrogel that adheres to the tissue of the implant area and is reabsorbed within 7 days of implantation according to the manufacturer’s specifications.

*Progrip™* (Medtronic, Minneapolis, MN, USA). A self-gripping mesh composed of PP with reabsorbable polylactic acid microhooks that provide immediate fixation to the implant tissue and are reabsorbed within 12–15 months.

*Surgipro™ CA*. PP mesh (Medtronic) fixed with 150 µl of CA adhesive (Ifabond, Peters Surgical, Bobigny, France) was applied as a spray with a diffuser (Sample Spray 2.5; Sunbox Distribution, Barcelona, Spain).

### Surgical technique and sample collection

Before surgery, the animals received an analgesic dose of 0.05 mg/kg buprenorphine (Buprecare, Divasa Farmavic, Barcelona, Spain). Subsequently, general anesthesia was induced with ketamine (20 mg/kg, Imalgene, Merial, Barcelona, Spain) and xylazine (3 mg/kg, Xilagesic 2%, Calier, Barcelona, Spain) administered intramuscularly. Using a sterile surgical technique, an approximately 7-cm-long incision in the skin was made in the right lateral side at a 3 cm distance from the abdominal linea alba.

The skin was dissected from the abdominal wall to leave a surgical field (6 × 4 cm) that allowed for the excision of the internal and external oblique muscle, leaving intact the transverse muscle, fascia transversalis and parietal peritoneum. In a random manner, each partial defect was repaired using 6 × 4 cm meshes. No additional fixation methods described in the study group were used. The subcutaneous tissue and skin were closed with a running 3/0 suture (Ethicon, Somerville, NJ, USA). After surgery and once daily for the first 3 days postsurgery, the animals received an analgesic dose of buprenorphine. Following surgery, the animals were monitored daily to detect any signs of postsurgical complications (warmth, dehiscence, abscess formation, etc.). 14 and 90 days post-implantation, the animals were anesthetized again and euthanized with an overdose of intravenous sodium pentobarbital (150 mg/kg, Dolethal, Vétoquinol, Lure, France).

At the end of the established study times, the dimensions of the meshes were measured and photographed before excision from the abdominal wall to determine whether there was a reduction in the implant area with respect to the original size meshes (% mesh shrinkage). The percentage of shrinkage was determined by computerized image analysis using ImageJ software (https://imagej.nih.gov/ij).

From each of the animals, skin and subcutaneous tissue were removed and specimens that included both mesh and surrounding tissue were collected. Two or three (depending on the degree of implant shrinkage) tissue and mesh samples of 1.5 cm were reserved for biomechanical tests. The rest of the sample was used for morphological and immunohistochemical studies.

### Morphological analyses

For the light microscopy (LM) analyses, samples were fixed in F13 solution and embedded in paraffin, cut into 5-mm-thick sections by microtome and stained according to the standard procedure for hematoxylin–eosin, Masson trichrome and Sirius red. Finally, the samples were mounted using Canada balsam and then visualized with a Zeiss Axiophot microscope (Carl Zeiss, Oberkochen, Germany). Sirius red staining was observed under polarized light. This technique is based on the orientation and interaction between the sulfone groups of the dye, the amine groups of lysin and hydrolysin, and the guanidine groups of arginine in the collagen fibers, thus producing different colors depending on the type of collagen. Type I collagen (mature) appears as a reddish-orange stain, while type III collagen (immature) takes on a yellowish-green shade.

Tissue fragments were fixed in 3% glutaraldehyde for scanning electron microscopy (SEM), dehydrated in a graded series of ethanol, desiccated with carbon dioxide using a Polaron CPD7501 critical point dryer (Fisons Instruments, Glasgow, UK), gold–palladium coated, and examined under a JSM-IT500 InTouchScope™ scanning electron microscope (JEOL Ltd., Tokyo, Japan).

### Immunohistochemical analyses

For the immunohistochemical study, the macrophages number per microscopic field was determined after labeling with the rabbit monoclonal antibody RAM-11 (DAKO M-633, Santa Clara, CA, USA; dilution 1:50) using a conventional protocol for avidin–biotin procedures. Biotinated IgG was used as a secondary antibody to amplify the signal and then labeled with avidin. To detect the antigen–antibody reaction, an alkaline phosphatase-fast red reaction was used, followed by final cell nuclei staining with hematoxylin. RAM-11-positive cells were counted in 10 microscopy fields per sample using a Zeiss Axiophot microscope (magnification 200×).

### Biomechanical assay

For mechanical testing, the strength of mesh fixation to the underlying tissue was evaluated at 14 and 90 days using the lap-shear method on freshly harvested samples immediately after slaughter according to the standard F2255-05 (standard test method for strength properties of tissue adhesives in lap-shear by tension loading). The strip length, width and thickness were determined with a digital caliper. Three measurements at different locations were obtained for each sample to ensure sample homogeneity. One short edge of the mesh was freed from the fascia at a length of 1 cm and inserted between the instrument’s upper clamps, thus leaving 3 cm adhered to the tissue (fixation testing area). The bottom clamp clasped the abdominal wall tissue. Tests were performed under displacement control on an INSTRON 3340 tensiometer (Instron Corp., Norwood, MA, USA) with a 1 kN full-scale load cell. The applied displacement rate was 5 mm/min until failure. The load (maximum load sustained) and displacement at failure were recorded. To compare the adhesion strength of the different groups, we defined two new variables: *tensile strength*, which was computed as *P* [N/mm] = load [*N*]/width [mm]; and *failure* s*tretch*, which was computed as *λ* [−] = *L*_0_ + Δ*L*/*L*_0_, where L_0_ is the initial distance between the clamps and Δ*L* is the displacement.

### Statistical analysis

The statistical comparison of the data (mean and standard deviation) was performed using Student’s paired *t* test (in the case of a normal distribution) or Wilcoxon’s test (in the case of a nonnormal distribution). The test of normality was performed by the Shapiro–Wilk test, and *p* values less than 0.05 were considered significant (no normal distribution). Statistical analyses were performed using MATLAB 2010 (The MathWorks Inc., Natick, MA, USA).

## Results

### Macroscopic observations

There were no complications related to the anesthesia or surgical procedures. No signs of surgical site or mesh infection were observed in any of the three study groups; therefore, all animals were included in the study.

At 14 days post-implantation, seroma was evident in the Adhesix implants (four of six implants: 4/6) and some Surgipro CA implants (2/6). All of them showed a thin fibrous capsule. Contrary to these observations, animals from the Progrip group did not exhibit any seroma formation. At 90 days post-implantation, seroma formation was not evident in any of the groups.

Adhesix exhibited poor integration into host tissue after 14 days of implantation. Three of six implants (3/6) showed small areas where the mesh was detached from the host tissue. Such detachment was not observed in any of the Progrip and Surgipro CA samples, where the meshes presented good tissue integration.

**Fig. 2:**
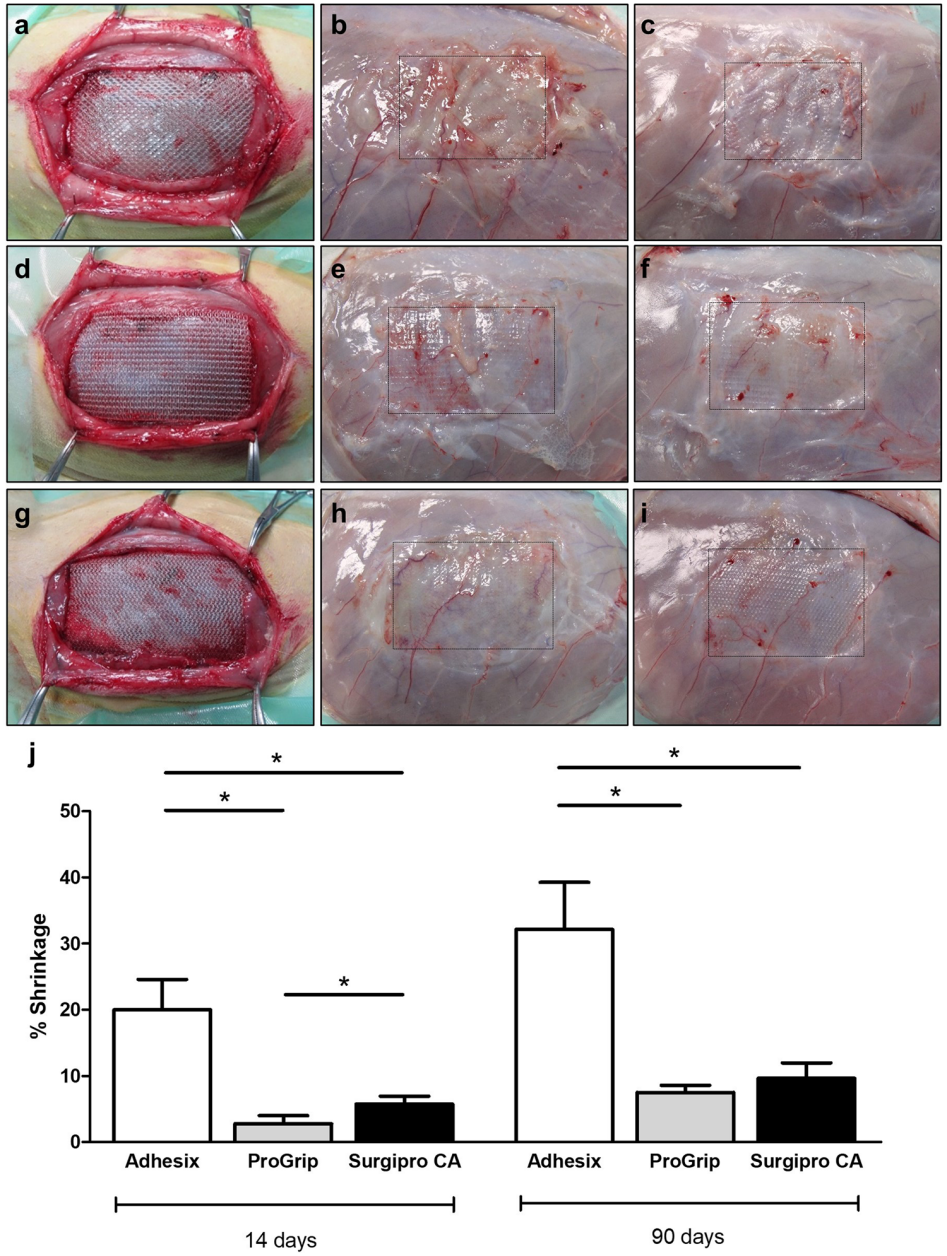
Macroscopic appearance of the Adhesix (**a–c**), Progrip (**d–f**) and Surgipro (**g–i**) meshes after implantation into the experimental animals (**a**, **d**, **g**) at 14 (**b**, **e**, **h**) and 90 days (**c**, **f**, **i**) after surgery. The implant contour is indicated with a black dotted line. Percentage of shrinkage at the different study times (**j**). *p < 0.05 (color figure online)

At 90 days postoperative, poor tissue integration was also observed in two of six (2/6) Adhesix implants while good integration into the host tissue was observed for the Progrip and Surgipro CA implants (Fig. 2a).

Prior to sample collection, photographs were taken and the prosthetic material was measured to determine the shrinkage in the implant area after the repair process.

At 14 days, the macroscopic results and implant area measurements revealed that the Adhesix implants showed a significantly higher degree of shrinkage than the Progrip and Surgipro CA implants (*p* < 0.05). The lowest values of shrinkage were observed for Progrip, and the values were significant relative to that of Surgipro CA (*p* < 0.05). At 90 days, there was a slight increase in the shrinkage percentage in all groups, although Adhesix again showed values significantly higher than that of the other two groups (*p* < 0.05) (Fig. 2b).

### Morphological analysis

**Fig. 3:**
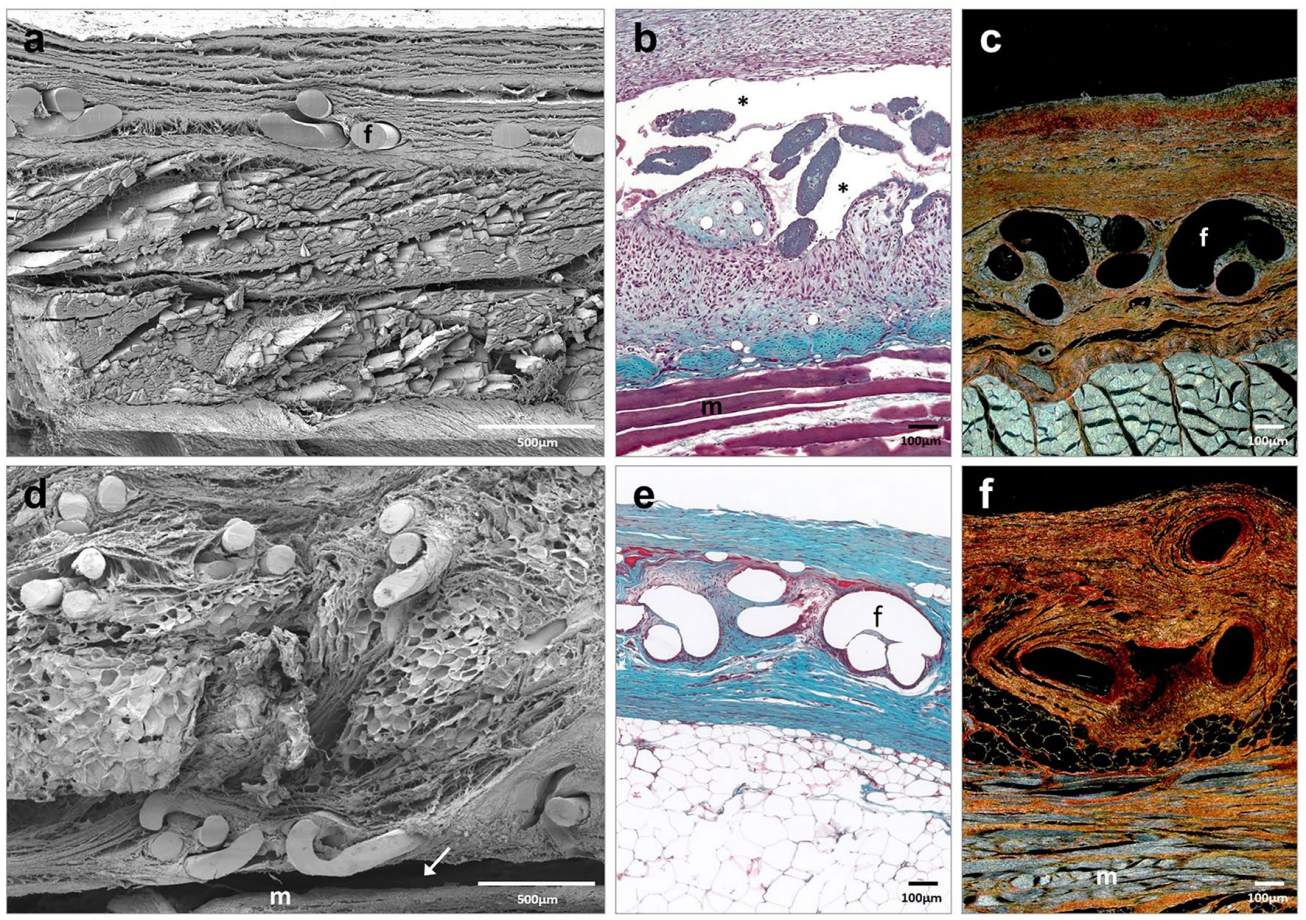
Scanning electron microscopy (**a**, **d**) (× 500; scales: 500 μm) and light microscopy images (Masson trichrome (**b**, **e**) and Sirius red staining (**c**, **f**), × 100; scale: 100 μm) of Adhesix mesh at 14 days (**a–c**) and 90 days (**d–f**) post-implantation. Sirius red staining shows collagen I (mature) in red and collagen III (immature) in yellow. Symbols: **f** mesh filaments; (m) muscle; (→) poor integration; and (*) area of seroma (color figure online)

At 14 days, the histological findings of the Adhesix samples showed the presence of PP filaments; however, the hydrogel was completely reabsorbed. In some detached zones, the LM and SEM microscopy images allowed for the validation of macroscopic observations regarding the lack of mesh integration and seroma formation beneath the prosthetic materials (Fig. 3a–c).

Scar tissue was composed of collagen fibers, predominantly type III collagen (Table 1), and inflammatory cells, including macrophages and foreign body giant cells, appeared around filaments and more numerous in areas associated with seroma.

**Table 1:** Semiquantification of collagen I (mature) and III (immature) expression in the neoformed tissue detected in the implant area in the different study groups

	Collagens			
	14 days		90 days	
	Type I	Type III	Type I	Type III
Adhesix	+	+ + +	+ + +	+ +
ProGrip	+	+ + +	+ + +	+ +
Surgipro CA	+ +	+ +	+ + +	+ +

The scale used for semiquantification was as follows: + , minimum staining (< 25%); +  + , moderate staining (25–50%); +  +  + , strong staining (50–75%); and +  +  +  + , maximum staining (> 75%)

At 90 days post-implantation, the inflammatory process had resolved, repair connective tissue richer in type I collagen was observed around the prosthetic filaments (Table 1), and the space between the mesh and underlying fascia was mostly occupied by adipose tissue (Fig. 3d–f).

**Fig. 4:**
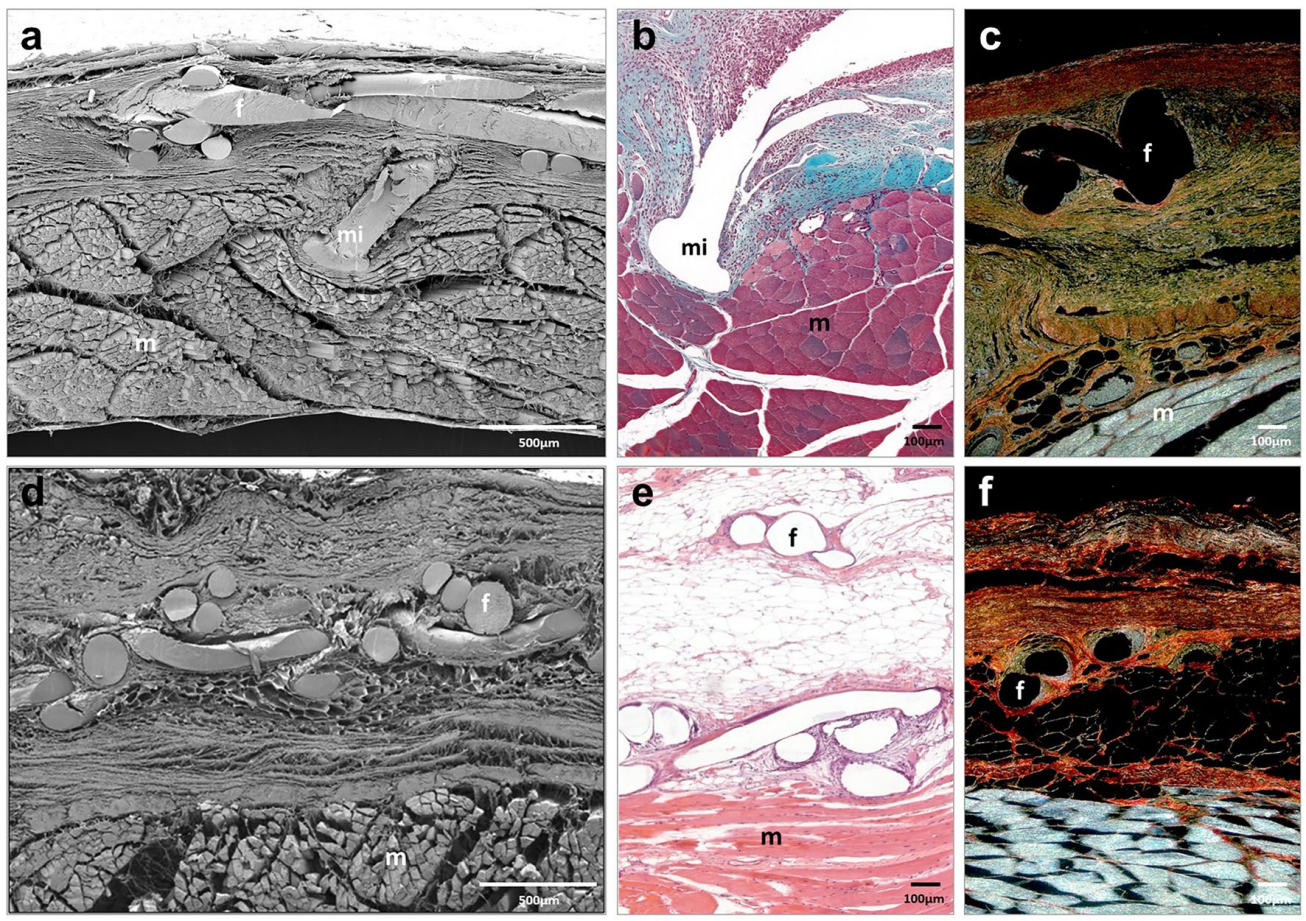
Scanning electron microscopy (**a**, **d**) (× 500; scales: 500 μm) and light microscopy images (Masson trichrome (**b**), hematoxylin and eosin (**e**) and Sirius red staining (**c**, **f**), × 100; scale: 100 μm) of Progrip mesh at 14 days (**a**–**c**) and 90 days post-implantation (**d–f**). Symbols: **f** mesh filaments; (m) muscle; and (mi) microhook

At 14 and 90 days post-implantation, Progrip implants were fully integrated into the host tissue. Neoformed connective tissue showed the same distribution as Adhesix implants. Blood vessels, fibroblasts and type III collagen fibers (Fig. 4; Table 1) were observed. Polylactic acid microhooks were observed at all study times without signs of evident absorption. Macrophages and inflammatory cells, mostly monocytes and polymorphonuclear cells, were observed around the Progrip filaments and microhooks. Similar to the Adhesix implant, great development of adipose tissue beneath the prosthetic material was observed at 90 days postimplant (Fig. 4d–f).

**Fig. 5:**
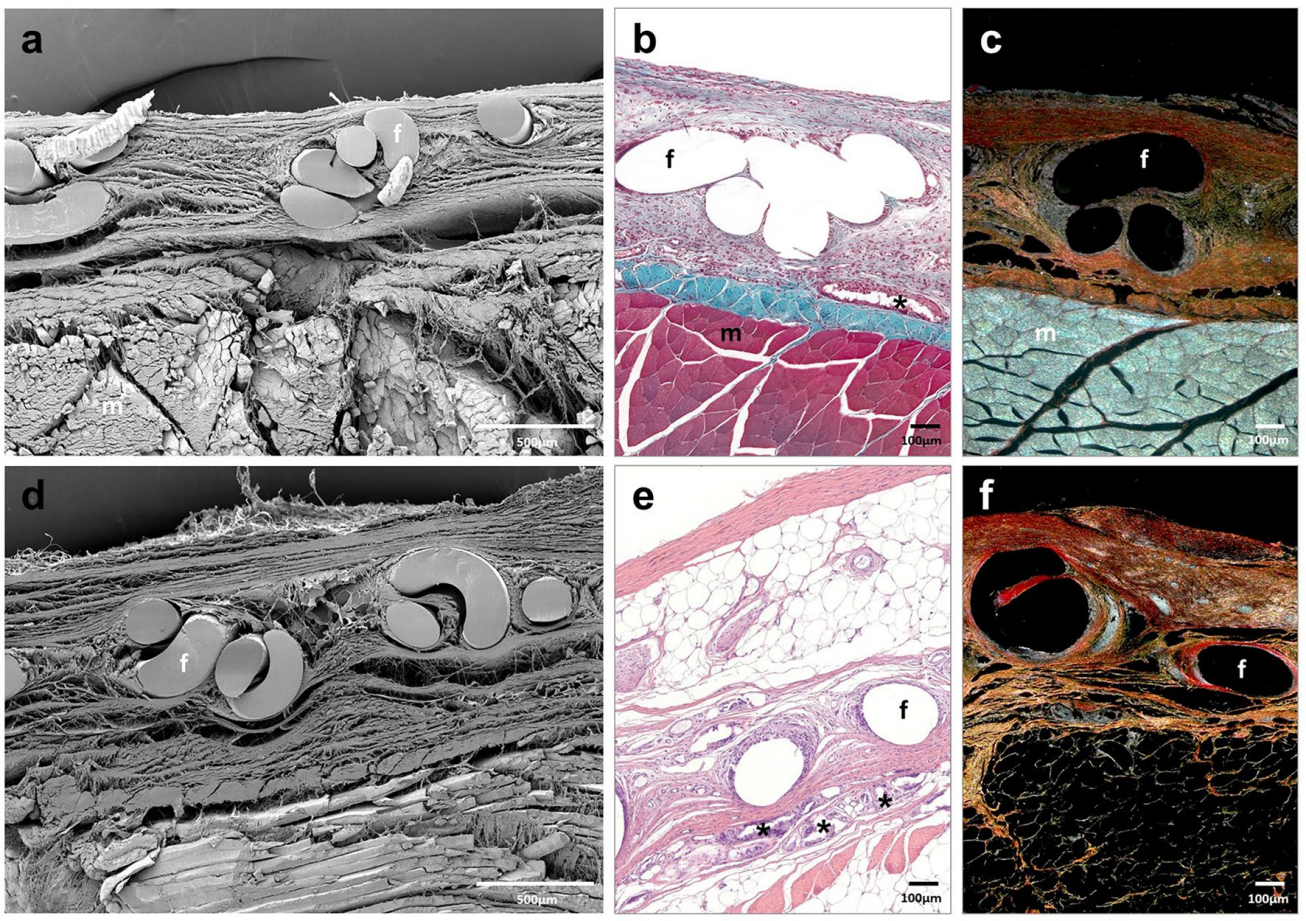
Scanning electron microscopy (**a**,** d**) (× 500; scales: 500 μm) and light microscopy images (Masson trichrome (**b**), hematoxylin and eosin (**e**) and Sirius red staining (**c**,** f**), × 100; scale: 100 μm) of Surgipro CA mesh at 14 days (**a–c**) and 90 days post-implantation (**d–f**). Symbols: **f** mesh filaments; (m) muscle; and (*) tissue adhesive

In the Surgipro CA group, remnants of tissue adhesive were observed to be zones with different sizes dispersed between polypropylene filaments at 14 and 90 days (Fig. 5). Scar tissue was mainly distributed around the mesh filaments. However, in this group, this tissue was composed of larger amounts of collagen type I (Table 1). Fibroblasts, macrophages and other inflammatory cells were observed around filaments and adhesive margins. This behavior could be related to the nondegradation of the adhesive. At 90 days, a less important inflammatory reaction could be observed than in the short term, although it remained around the rest of the tissue adhesive. The 90-day scar tissue was similar to that of the previous groups, with adipose tissue accumulation (Fig. 5d–f).

### Macrophage response

**Fig. 6:**
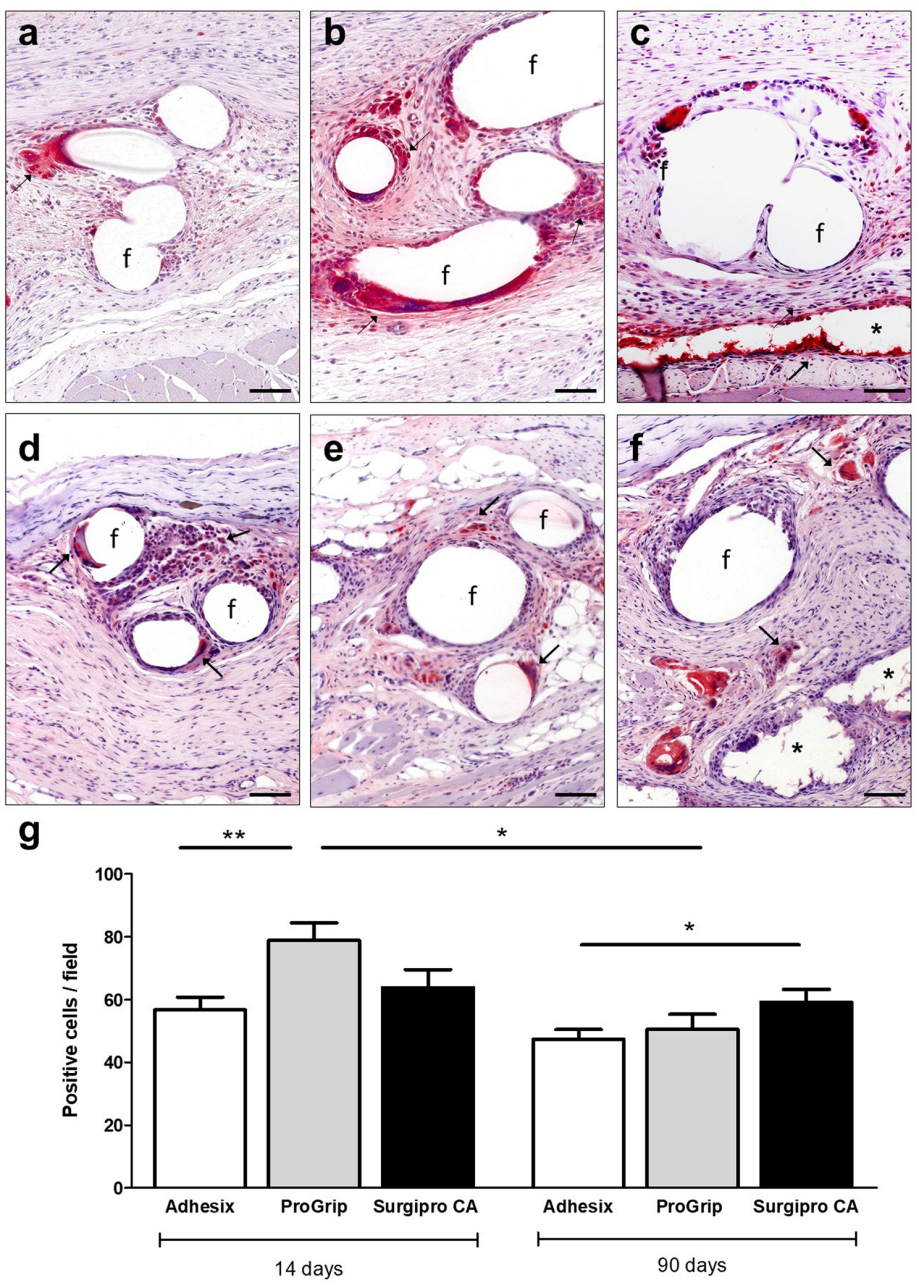
Macrophage response. Micrographs representative of Adhesix (**a**,** d**), Progrip (**b**,** e**) and Surgipro CA (**c**,** f**) meshes, demonstrating the distribution of RAM-11-positive cells in red around the prosthetic filaments (immunohistochemistry, × 200; scales: 100 μm) at 14 days (**a-c**) and 90 days (**d-f**). Symbols: **f** mesh filaments; (→) labeled macrophages and (*) tissue adhesive. Macrophage-positive cells per field of the different meshes at the different time points (**g**). *(*p* < 0.05) and **(*p* < 0.01)

The macrophage response to the implants was assessed by examining RAM-11-positive cells in the implant area. The results showed the presence of RAM-11-positive cells mainly distributed around the mesh filaments in all the groups (Fig. 6). Macrophages could also be observed in the Surgipro CA group delimiting the area occupied by the tissue adhesive (Fig. 6c, f) and in the Progrip group around the polylactic acid microhooks (Fig. 6b, e).

At 14 days, macrophage counts were greater in the Progrip group than the Adhesix and Surgipro CA groups, and the differences were only statistically significant with Adhesix (*p* < 0.01) (Fig. 6g).

At 90 days, the macrophage response decreased with respect to the response in the short term in all three groups; however, the differences were only significant in the Progrip group (*p* < 0.05*)*. At this time point, Adhesix showed significant differences with respect to Surgipro CA (*p* < 0.05*)* (Fig. 6g).

### Biomechanical study

At 14 days post-implantation, the biomechanical analysis was not possible because 50% of the meshes in the Adhesix group were partially detached from the host tissue and 66% presented seroma formation beneath the prosthetic material.

In the lap-shear methods, nonlinear behavior was observed regardless of the fixation method studied. In the statistical analysis, all fixation methods showed a normal distribution (*p* > 0.05).

**Fig. 7:**
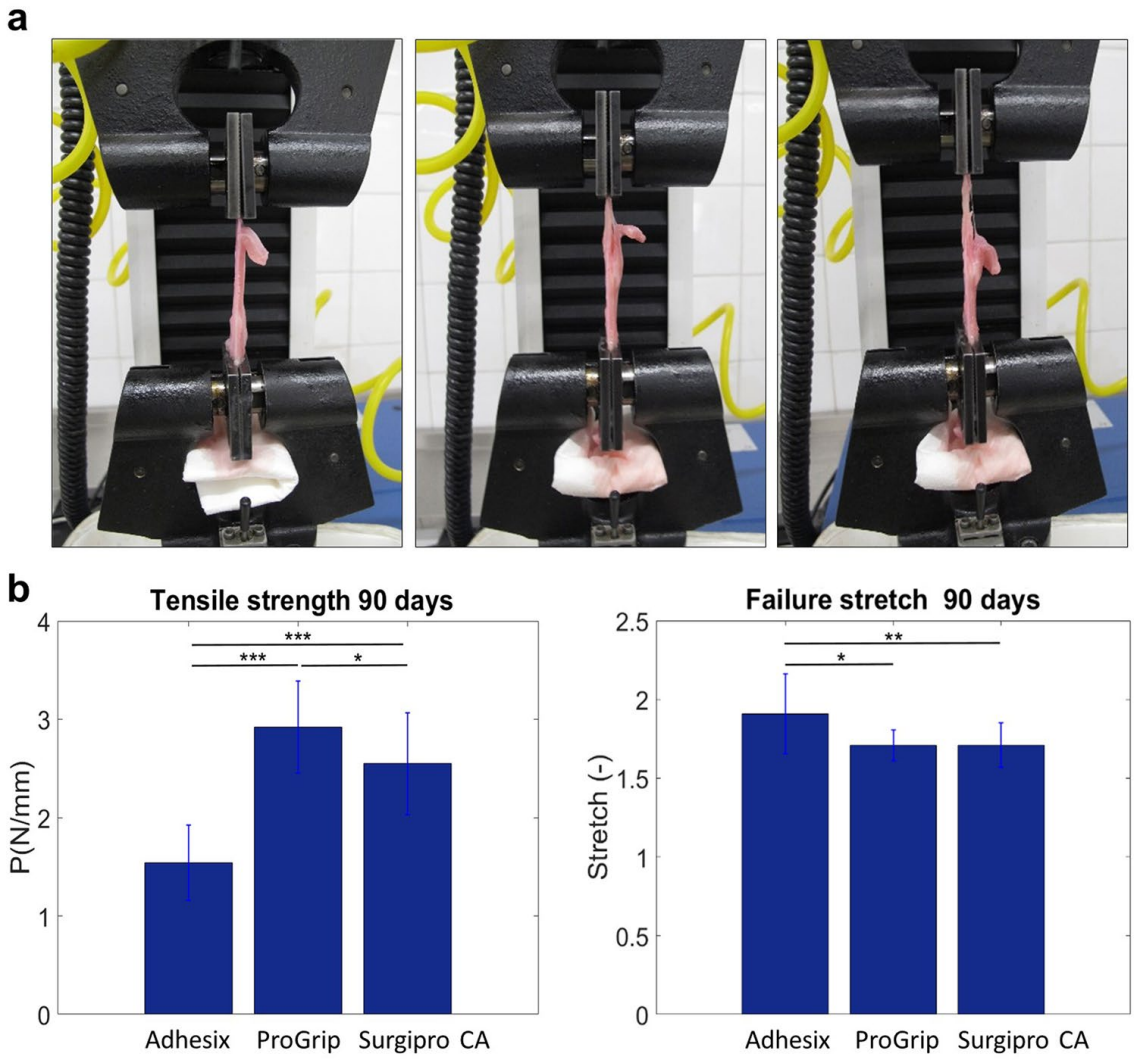
Biomechanical assay sequence showing grip displacement in the lap-shear method (**a**). Mean tensile strength and failure stretch values (± standard deviation) for the different fixation groups at 90 days (**b**). *(*p* < 0.05); **(*p* < 0.01)*;* and ***(*p* = 0.0001)

At 90 days, significant differences in tensile strength and failure stretch values were observed between all fixation methods (*p* < 0.05). The highest tensile strength values were recorded in the Progrip group (2.920 ± 0.471 MPa), and the lowest values were obtained in the Adhesix group (1.542 ± 0.382 MPa). Statistical differences between both groups were observed (*p* = 0.0001). Failure stretching showed significant differences between the Adhesix group and the rest of the groups (Fig. 7b).

## Discussion

In hernia repair processes on the abdominal wall, reinforcement with prosthetic materials in the form of meshes is considered the gold standard. In those cases in which the hernia defect is closed, mesh fixation would not be necessary, but otherwise prosthetic fixation is recommended to prevent the mesh from displacement in the early postoperative course. Currently, there are surgeons who leave these defects open ensuring that a 5-cm mesh overlap is adequate, however, mathematical confirmation has shown that more important defects require greater overlaps to minimize the risk of migration and recurrence [29].

The fixation, when necessary, of these meshes with traumatic devices, such as sutures or tacks, is recognized as a causal factor in the development of postoperative pain and directly related to the damage or entrapment of nerve endings in the receptor tissue, which largely cause postoperative neuralgia in patients after hernia surgery.

Alternatively, either natural or synthetic adhesives applied to the receptor tissue bed are used as a common alternative for mesh fixation [30–32]. Some of these medical adhesives have already been evaluated by our research group in preclinical studies with very promising results, especially using long-chain CA, which considerably reduces tissue toxicity and shows quite good biological and mechanical behavior in the hernia repair process [24, 33]. In the present experimental study, we used one of these tissue adhesives, namely, an n-hexyl CA monomer, as a control to compare its biological and mechanical behavior with that of self-fixing meshes, which constitute another very good alternative for atraumatic abdominal prosthetic fixation. This group includes meshes equipped with systems that fix the mesh to the receptor tissue either through “grip” type devices [14, 34] or through adhesive hydrogels included with the meshes [13, 16, 27, 28, 35, 36].

We designed a biological and mechanical study of the behavior of the two self-fixing meshes and selected two prostheses with different fixation mechanisms, the self-adhesive hydrogel mesh (Adhesix) and the self-gripping (ProGrip®), which both avoid the use of additional fixation and have demonstrated a significant reduction in the incidence of postoperative pain at the clinical level [26–28].

Most of the published clinical studies have analyzed short-term complications, chronic pain and recurrence rates as their primary outcomes [35, 37, 38]. However, few publications, none of them related to Adhesix, have examined the mechanical behavior and host tissue response in preclinical animal models using the aforementioned self-fixing devices. In our study, the experimental animal was the New Zealand white rabbit, for which we have extensive experience [39]. The experimental model is an acute partial abdominal wall defect with a sufficient size to carry out both morphological and mechanical studies. The study times of 14 and 90 days were designed to corroborate the short- and medium-term behavior of the materials used, with special emphasis on their mechanical response.

At the macroscopic level, we observed that in the short term, Progrip and Surgipro CA showed good integration within the host tissue versus Adhesix, with 4 of the 6 implants showing seroma and 3 implants exhibiting mesh that appeared detached from the recipient tissue, which explained why this study time could not be included in the biomechanical studies and only the medium term of 90 days was included. These indications suggest that fixation with the resorbable hydrogel formed by polyethylene glycol and polyvinylpyrrolidone is not strong enough and impairs the tissue integration process. Indeed, if the hydrogel interferes by obstructing the pores of the mesh, can prevent tissue integration of the prosthetic material. In the case of the Progrip this does not happen, that is why it is the one that best integrates into the receptor tissue. Prospective clinical studies have shown low rates of postoperative complications following inguinal hernia repair with Adhesix, which is inconsistent with our results [35, 40]. A 3-year retrospective study also showed that this mesh is associated with low chronic pain, recurrence and postoperative complication rates [27].

In contrast, experimental studies in rats have shown that this hydrogel fixation mesh has a greater probability of dislocation from its implantation site than mechanical grip fixation, which is consistent with our results [41], and such dislocation would translate into recurrences in clinical practice. The lack of integration in Adhesix was also observed in our case three months after implantation in 2 of the 6 samples. Good adhesion of Progrip and Surgipro CA was observed in this same study time, and no presence of seroma was seen in either group.

Our implants also showed a significantly higher degree of mesh shrinkage in the Adhesix group than in the rest of the groups both in the short and long term, reaching important values close to 30% at 90 days. Previous authors [41] have also shown this problem of prosthetic contraction but to a lesser extent. In this case, mesh shrinkage was below statistical significance and PP reached 5% in some implants, which is still considered not problematic. Incorporation of the Adhesix prosthesis was evaluated as excellent both macroscopically and microscopically in laparoscopic surgery performed in pigs [13], with a significant amount of fibrosis thickness and no real shrinkage; however, 16% of cases showed mild folding.

Referring to the biomechanical properties, at 90 days, the highest tensile strength values were recorded in the Progrip group and the lowest ones were obtained in the Adhesix group, with strong significant differences observed between both groups. However, the maximum significant value of failure stretch was observed in the Adhesix group compared to the other two groups, thus indicating that the fixation in the Adhesix group was more inefficient, with a higher elongation and a lower tension at failure meaning that the system was very deformable and the fixation was weaker.

Few studies have analyzed the biomechanical properties of self-fixing meshes, and none of these studies have focused on Adhesix.

An experimental study confronting Progrip with fixation with a stapler or fibrin glue in a rat model noted that the self-fixing prosthesis showed substantially stronger strength of incorporation in muscle tissue compared with the other fixation systems both in the short and medium term at 5 days and 2 months, respectively [34]. Another experimental study [7] on mongrel swines that compared fibrin sealant and Progrip with a self-fixing mesh also made of PP coated with gelatin, which undergoes intra- and intermolecular crosslinking catalyzed by microbial transglutaminase (LifeMesh™) after contact with water, showed unexpected results for this mesh, which that significantly increased the strength of the mesh-fascia interface. Biomechanical testing in this case was performed 10 min after implantation of the mesh, which is considered a very early time point that does not allow for tissue growth and prosthetic integration. The authors explain that this crosslinking gelatin technology represents a relatively strong adhesive [7].

An ex vivo porcine and bovine model used to assess gel-coated ProGrip for dislodgement shear forces before and after dissolving the gel showed that this technology significantly decreased the attachment forces of the ProGrip mesh and did not impair the self-gripping properties after dissolving [42].

Regarding the histological examination in our study, the Adhesix samples corroborated the macroscopic observations regarding the lack of integration of the mesh and the presence of seroma below the prosthetic material. The rest of the meshes presented good integration with healing tissue that matured in the long term, was richer in type I collagen and had an increased adipose component. The complete disappearance of the adhesive hydrogel of the Adhesix was observed at 14 days, while the hooks of the Progrip were still present, which led to a higher macrophage response in this last study group. Microhooks are reabsorbed within 12–15 months, whereas the hydrogel was reabsorbed within 7 days of implantation. Other authors [34] have histologically corroborated that the microhooks of the self-gripping mesh were also generously surrounded by tissue and embedded to a depth of approximately 0.5 mm in the deeper tissue. This superior tissue integration was reflected in the biomechanical results, with the highest tensile strength values recorded in the Progrip group [34].

The Progrip mesh showed the greatest macrophage response in the short term; however, in the long term, RAM-11-positive cells decreased considerably in all of our study groups, with a significant decrease in the Progrip group, although the microhooks of resorbable material were still present at this time of study. Hollinsky et al. [34] also showed that the inflammatory reaction in Progrip was considerably more severe in the short term than after 2 months and observed fewer lymphoid, plasma, macrophage and granulocyte cells in the repair tissue, and these findings are consistent with our results. Histological examinations of other studies comparing the two self-fixing meshes included here have also found a slightly pronounced foreign body reaction represented by macrophages and foreign body giant cells in all groups [41]. Other experimental studies that have histologically observed the effects of Adhesix [13] have reported that at one week and one month, incorporation of the prosthesis within the abdominal wall was complete in all cases, with the development of a fibrous and inflammatory reaction limited to the mesh and its close periphery; however, none of these studies showed corresponding images.

Obviously, certain limitations were observed in our study. For example, the animal model cannot be easily transferred to the clinic and may not be generalized to the human population. Thus, although good results were shown by these two self-fixing meshes in the clinical setting, conclusions about the behavior of these materials should be made with caution. The high seroma index found in Adhesix could be related to tissue manipulation and the existence of areas where the self-adhering gel coating had not been activated by humidity and body heat; moreover, the absorbance of water from the adjacent tissue and loss of adhesive properties in these areas could have translated into a lack of integration due to mesh displacement and shrinkage [43]. Another limitation that should be taken into consideration is that the onlay model can allow for some movement of the mesh [41].

Taking into account all these considerations and the results obtained in our study, we can conclude that PP meshes with mechanical microgrip self-fixation show better biological and mechanical behavior than hydrogel fixation in our model of abdominal hernia repair in rabbits.

## Data Availability

Data presented in this study are available in the manuscript.
